# Adaptive Precision Nutrition: Integrating Dynamic Phenotyping, Multi-Omics, Artificial Intelligence, and the Allostatic Viability Principle

**DOI:** 10.3390/nu18203373

**Published:** 2026-10-14

**Authors:** Alexander G. Haslberger

**Affiliations:** Department of Nutritional Sciences, Faculty of Life Sciences, University of Vienna, Josef-Holaubek-Platz 2 (UZA II), 1090 Vienna, Austria; alexander.haslberger@univie.ac.at

**Keywords:** precision nutrition, personalized nutrition, functional foods, multi-omics, artificial intelligence, phenotypic flexibility, adaptive capacity, allostasis, viability, nutrigenomics

## Abstract

Precision nutrition increasingly combines functional foods, multi-omics, digital phenotyping, and artificial intelligence, yet the incremental decision value of additional data layers remains uncertain. This critical narrative review and hypothesis-generating conceptual proposal examines evidence on personalized nutrition, dynamic response phenotyping, functional-food heterogeneity, multi-omics integration, and AI-supported decisions. Personalized advice can improve dietary behavior and selected cardiometabolic outcomes, whereas the additional clinical benefit attributable specifically to elaborate molecular profiling has been inconsistent. Dynamic challenge studies suggest that response amplitude, timing, coordination, recovery, and conditional repeatability may provide information beyond static profiles, but reproducibility is required before these features can be treated as person-specific traits. The Allostatic Viability Principle (AVP) is proposed as a conceptual physiological and decision framework rather than a validated predictor or biomarker. Its intended contribution is to organize existing approaches around viable, context-appropriate regulation, proportional use of additional measurements, explicit uncertainty, an outcome hierarchy that does not equate molecular change with health benefit, and a response-and-revision loop. Whether this organization improves decisions or outcomes beyond high-quality conventional or adaptive personalized nutrition remains an empirical question. The framework is therefore presented as a testable structure for determining when additional individual-level information is reproducible, relevant, and sufficiently decision changing to justify use.

## 1. Introduction

Precision nutrition and multilayer omics are developing quickly. Genomic, epigenomic, metabolomic, and microbiome data can now be combined with clinical, dietary, behavioral, and digital information at a scale that was difficult to imagine a decade ago. The harder problem is interpretation. Measurement variability, temporal change, causal ambiguity, and disagreement between data layers still limit reproducible translation into individual recommendations. Nutrition science is moving beyond population-level advice, but the key methodological question remains which measurable differences genuinely improve a decision [1,2,3,4,5,6,7,8]. Responses to food depend on baseline metabolic state, age, genetics, epigenetic regulation, microbiome function, medication, physical activity, sleep, psychosocial context, and the food environment. Functional foods add further complexity because the effect of a polyphenol, fermentable substrate, probiotic, fatty acid, or other bioactive component depends on absorption, biotransformation, and the regulatory state of the host as well as on dose. It is no longer enough to show that people differ; the relevant differences must also be reproducible, actionable, and worth measuring.

The evidence is less straightforward than the technological promise sometimes suggests. In Food4Me, personalized advice improved dietary behavior compared with conventional generalized guidance, but adding phenotypic and genotypic information did not produce a simple gradient of benefit with increasing data density [2]. Randomized and systematic-review evidence supports a potential effect of personalized advice on dietary behavior, but the field remains heterogeneous and certainty is often limited [9,10]. Null or mixed findings should not be interpreted as an intrinsic failure of multi-omics: they may reflect intervention intensity, adherence, comparator quality, population selection, implementation, or the choice of primary endpoint. Four questions should therefore remain separate throughout the field: whether receiving personalized advice improves outcomes; whether additional biological or digital data provide incremental value beyond simpler models; how accurately a model predicts a response; and whether using that model to select an intervention causally improves clinical or functional outcomes.

Randomized trials illustrate this distinction. A machine-learning diet designed to reduce predicted postprandial glycemic responses did not produce greater weight loss than an established low-fat diet in adults with obesity and abnormal glucose metabolism [3]. The ZOE METHOD trial evaluated a bundled personalized program that combined food characteristics, postprandial responses, microbiome features, health history, and programmatic support; LDL-cholesterol and fasting triglycerides were both primary outcomes. Triglycerides decreased significantly relative to control, whereas the between-group LDL-cholesterol difference was not significant; several secondary outcomes also improved [4]. The trial therefore supports the potential utility of the bundled program but does not isolate the causal contribution of any single biological personalization layer. Similarly, prediction models derived from one cohort require external validation, recalibration, or retraining when populations, foods, devices, or measurement systems change.

PREDICT 1 demonstrated substantial inter-individual variability in postprandial triglyceride, glucose, and insulin responses and developed multimodal prediction models, while also illustrating that prediction performance differs by outcome and data source [11]. The NIH Nutrition for Precision Health program extends this measurement-and-prediction architecture through controlled and free-living dietary studies, multi-omics, dietary assessment, contextual data, and dedicated AI infrastructure [7,12]. These programs provide realistic test beds for dynamic precision nutrition. The proposed AVP framework does not claim to replace their measurement architecture; its narrower question is whether a physiological target of viable regulation, a proportionality rule for adding data, and an explicit response-and-revision decision process improve what is done with such measurements.

Recent position papers and methodological reviews increasingly make these distinctions explicit. Precision nutrition is now discussed in terms of data fusion, measurement quality, temporal dynamics, behavioral feasibility, causal inference, external validation, and equity, not simply as an extension of nutrigenetics or multi-omics profiling [5,6,7,8,13,14]. This creates a genuine paradox: more biological layers can be measured than ever before, but decision-relevant certainty does not necessarily rise with them. Progress may depend less on maximizing inputs than on identifying which information changes a decision, with what degree of confidence, and whether the subsequent response supports the original prediction.

Earlier work in personalized nutrition and nutritional epigenetics already pointed in this direction by emphasizing that dietary effects are embedded in regulatory networks rather than determined by single markers [15,16,17]. Studies of lifestyle-sensitive blood-derived microRNAs and mitochondrial regulatory markers add a more recent example: molecular signatures can be modifiable and informative, yet still require longitudinal validation before they can support individual prescriptions [18,19,20]. The question taken up here is therefore broader than how to profile an individual. It is whether precision nutrition can incorporate intervention response, recovery, uncertainty, and revision into the phenotype itself.

The novelty claim is deliberately narrow. Repeated measurement, phenotypic-flexibility testing, adaptive personalized advice, N-of-1 designs, digital closed-loop systems, SMARTs, micro-randomized trials, and learning health systems all predate AVP [13,21,22,23,24,25,26,27]. AVP is not proposed as a new biomarker, challenge test, adaptive trial design, or predictive algorithm. It is proposed as a hypothesis-generating conceptual lens that combines a physiological criterion of viable regulation with an outcome hierarchy, proportional measurement, explicit uncertainty, and correctability. The empirical novelty to be tested is therefore whether this organization changes decisions in a beneficial way compared with high-quality existing approaches.

## 2. Scope and Approach of the Narrative Review

This article is a critical narrative review combined with a hypothesis-generating conceptual proposal. It is not a systematic review or meta-analysis. No prospective protocol was registered, and no formal risk-of-bias assessment, GRADE-style evidence grading, or pooled effect estimation was performed. The purpose is to synthesize decision-relevant evidence and methodological concepts and to make the proposed AVP-based architecture sufficiently explicit to be criticized and prospectively tested.

A targeted supplementary PubMed search was conducted on 3 October 2026 for publications dated 1 January 2021 to 3 October 2026. Six queries addressed personalized-nutrition trials, reproducibility of postprandial responses, phenotypic flexibility and adaptive frameworks, incremental decision value, functional-food response heterogeneity, and evidence syntheses. The queries yielded 804 record occurrences and 745 unique PMIDs after deduplication. Exact queries and counts are reported in Appendix A; retrieved identifiers and bibliographic records were retained. Named-study and DOI retrieval supplemented relevant recent publications and earlier landmark studies.

Human intervention studies, randomized or controlled trials, prospective cohorts, methodological studies, position papers, and major reviews were prioritized. Selection focused on reproducibility, person-by-food or person-by-intervention heterogeneity, incremental utility, null or discordant findings, adverse effects or burden, and clinical or functional outcomes. Nineteen publications were purposefully selected for targeted appraisal in this update; the broader narrative synthesis also retained relevant references from the previous version. All 19 abstracts were consulted, with selected full-text sections checked for the ZOE METHOD and Wu et al. studies; a full-text methodological appraisal of every selected publication was not performed. Evidence types and the causal inferences supported by each study were distinguished.

The original narrative search was not logged at record level and was not retrospectively reconstructed. The retained search queries, identifiers, and bibliographic records document the dated update; the summary here does not provide the complete query syntax needed for independent replication. This was not an exhaustive systematic review or an independent dual-reviewer eligibility screen. Retrieval counts should not be interpreted as counts of eligible studies. No language or species filter was applied during retrieval; non-human and unrelated records were not used in the targeted synthesis, and records not individually appraised were not counted as formal exclusions.

Generative AI-assisted manuscript preparation. During revision, OpenAI ChatGPT (OpenAI, San Francisco, CA, USA; used during revisions from 30 September to 8 October 2026) was used to assist with language editing, structural organization, reviewer-response synthesis, literature-search planning, and generation of alternative phrasings. It was not used to generate or alter original data and was not treated as a scientific source. Scientific statements remain grounded in the cited literature, and all AI-assisted text was reviewed and edited by the author.

## 3. Functional Foods as Context-Dependent Nutritional Challenges

Functional foods are often described through their bioactive constituents and molecular targets. That is useful mechanistically, but it does little to explain why the same exposure can have different effects in different individuals. For precision nutrition, it is more informative to view a functional food as a controlled perturbation of a biological system whose response depends on its pre-existing regulatory state. The outcome reflects food matrix, dose, and timing together with host metabolism, microbial biotransformation, previous dietary exposure, medication, circadian context, and behavior [28,29,30,31,32,33,34]. The relevant question is not only whether a compound can influence a pathway, but when that influence matters, in whom, and under which biological conditions.

Polyphenols make the problem clear. Their biological effects depend not only on parent compounds but also on extensive host and microbial transformation. Distinct microbial metabotypes can generate different urolithins, equol-related metabolites or resveratrol-derived products despite comparable intake, providing a plausible basis for responder subgroups [28,29]. At the same time, the exposure itself is often poorly characterized. A recent review of polyphenol-microbiome intervention studies found that comprehensive omics characterization of the administered food was uncommon, despite compositional variation introduced by cultivar, processing, and storage [30]. Precision nutrition therefore needs to characterize both the molecular substrate that was consumed and the metabolites actually produced in the individual.

The microbiome is an attractive integration layer, but it also shows why association alone is insufficient for personalization. Large cohorts reveal many links between habitual diet, microbial species, and microbial pathways, and longitudinal analyses indicate that numerous diet-microbiome associations persist over time [31,32,33]. Stability of association, however, does not show that deliberate manipulation of a microbial feature will produce the expected host benefit. The causal unit may be a community function, metabolic pathway, or host–microbial interaction rather than a single taxon. Microbiome-guided nutrition is scientifically much richer than it was a decade ago, but prospective evidence that microbiome-based recommendations improve outcomes remains limited and is affected by geography, medication, and reproducibility [32,33,34].

Microbial metabolites also illustrate regulation across layers. Short-chain fatty acids, ketone bodies and other small molecules connect dietary patterns, fasting, microbial metabolism and epigenetic regulation. Earlier work linked short-chain-fatty-acid-producing bacteria with epigenetic regulation of FFAR3 and described the microbial epigenome as a possible interface in metabolic regulation [16,17]. In a small randomized pilot trial combining probiotics with octacosanol, selected inflammatory transcripts and miRNA signals changed without parallel changes in anthropometric or lipid parameters [35]. These findings cannot isolate the contribution of the probiotics from that of the co-administered compound. That mismatch is informative. A molecular response can be genuine without amounting to a demonstrated health benefit, and precision nutrition needs to preserve that distinction.

Regulatory RNAs offer another example. A systematic review identified more than one hundred endogenous miRNAs associated with dietary exposures in intervention and observational studies, while also documenting substantial heterogeneity in tissues, intervention types and analytical strategies [36]. In a cohort of 305 participants, lifestyle and dietary patterns were associated with several capillary-blood miRNAs linked to inflammatory and metabolic pathways [18]. An exploratory three-miRNA biological-age model was likewise associated with lifestyle factors but still requires larger longitudinal validation [19]. Methylation and miRNA signals related to mitochondrial biogenesis can also change after combined exercise and dietary interventions [20]. These findings make regulatory molecules interesting dynamic intermediate phenotypes, but not stand-alone nutritional instructions.

For functional-food research, this has practical consequences. Trials should move beyond asking whether a product changes a selected biomarker. More informative designs would test whether baseline phenotypes predict response, whether the response is reproducible on re-exposure, whether a person-by-food interaction is demonstrable, and whether molecular changes agree with functional or clinically meaningful outcomes. Null findings, intolerance, symptom worsening, adverse effects, and burden are also decision-relevant outcomes and should not be treated as secondary to molecular sophistication. Functional foods are well suited to repeated-challenge designs because many can be administered under standardized conditions while responses are followed across molecular, metabolic, functional, and behavioral levels.

## 4. Dynamic Response Phenotypes in Human Nutrition

Most personalized-nutrition strategies still begin with a snapshot: genotype, fasting laboratory values, a microbiome sample, an epigenetic profile, or a metabolomic measurement. These can be informative, but they mainly describe the current state. Nutrition repeatedly perturbs metabolism, endocrine signaling, immune activity, and microbial function, so an individual’s response to a standardized challenge may reveal properties that are not visible at rest [21,37,38,39]. This is the basis of phenotypic flexibility: the coordinated capacity to respond to and recover from a physiological perturbation.

Dynamic metabolomics gives substantial support to this approach. The HuMet repository characterized 2656 metabolites in 15 healthy young men at repeated time points during glucose, lipid and mixed-meal challenges, prolonged fasting, exercise and cold exposure [39]. Earlier challenge studies had already shown that the human metabolome spans a much wider range under controlled perturbation than fasting samples suggest [40]. Repeated sampling captures amplitude, timing, pathway coordination and recovery kinetics, none of which is available from a single baseline value. Two people with similar fasting glucose or metabolite concentrations may therefore behave quite differently once challenged. The small, selected HuMet cohort limits direct generalization to broader populations.

Postprandial glycemia is particularly useful because it can be measured at high temporal resolution, although it also exposes the methodological difficulty of individualization. Responses to identical foods vary substantially between people and can be predicted from multimodal information [11,41]. For personalized advice, however, population heterogeneity is not enough: the relevant evidence is a reproducible person-by-food or person-by-intervention interaction under defined conditions. Meal order, previous diet, fasting interval, sleep, physical activity, medication, acute illness, sensor error, and ordinary day-to-day variation can all alter the apparent response. High repeatability under narrow laboratory conditions also does not establish transportability to free-living conditions [22,42]. The Zeevi et al. model [41] is an influential example of individualized postprandial prediction, but use beyond the original data-generating setting requires external validation and, where necessary, cohort-specific recalibration or retraining.

Recent findings require a balanced interpretation. Della Corte et al. [43] analyzed standardized glycemic tests in 382 healthy adults and found that individual glucose-reference responses scaled by the mean glycemic index of the food explained responses within test-retest variability, challenging strong claims of person-specific food effects. Other studies reported reproducible glycemic sensitivity [22] or food-response patterns associated with insulin resistance and beta-cell function [44]. These studies concern related but distinct questions: general glycemic sensitivity, context-dependent variation, and reproducible person-by-food interactions should be distinguished before response profiles guide dietary decisions.

Fluxomics extends dynamic phenotyping by using stable-isotope tracers together with kinetic or metabolic models to estimate pathway fluxes rather than relying on concentrations alone [45]. These estimates can reveal differences in substrate handling, synthesis, oxidation, exchange, or clearance that static concentrations may conceal. They nevertheless depend on tracer design, model structure, sampling, and assumptions; fluxomics does not directly observe pathway activity without inference and does not by itself establish the causal effect of a nutritional intervention. Its value is therefore strongest when dynamic estimates are interpreted alongside experimental design, uncertainty, and clinically or functionally relevant outcomes.

A dynamic view also changes what counts as a favorable response. Smaller is not necessarily better. Glucose, insulin, inflammatory mediators, stress hormones, and substrate oxidation are expected to move under appropriate conditions. What matters is whether the response is proportionate to the challenge, coordinated across relevant systems, and followed by effective recovery. The aim is not to flatten every excursion, but to assess regulated change more physiologically [21,37,38,39,40,45].

### 4.1. Identifying Person-Specific Responses Under Defined Conditions

Repeatability must be conditional rather than absolute. The same named food is not the same exposure when the food matrix, dose, co-ingested nutrients, timing, previous diet, or fasting interval differs. Some factors should therefore be standardized for a defined challenge, whereas others should be prespecified, recorded, and modeled because they may be part of the dynamic phenotype rather than nuisance noise. For a mixed-meal challenge, minimum standardization would usually include meal composition and dose, timing, fasting interval, sampling schedule, and measurement platform. Prespecified contextual modifiers may include sleep duration and timing, recent physical activity, medication, acute health status, menstrual or menopausal state where relevant, ambient or seasonal conditions, and major recent weight change.

Conceptually, an observed response can be decomposed into a population-average component, stable person-specific effects, food or intervention effects, prespecified context effects, interactions among these components, stochastic within-person variation, and measurement error. In notation, a repeated response Y may be represented as: Y = mu + person + food/intervention + context + person × food + person × context + food × context + residual variation + measurement error. Mixed-effects or hierarchical models are natural tools for this decomposition because repeated observations are nested within people and can estimate partial pooling, interaction variance, and uncertainty. Separating residual biological variation from measurement error requires technical replicates or external reliability information; they are not independently identifiable from ordinary repeated observations alone. The specific model should be chosen for the estimand and sampling structure rather than because hierarchy is intrinsically preferable.

Replicate crossover designs, repeated N-of-1 designs, and repeated standardized challenge tests are therefore more informative than a single pre/post comparison for responder classification. Useful descriptive quantities include within-person SD or CV, ICC, repeatability or reproducibility coefficients, and limits of agreement, but none answers every question. ICC is sensitive to between-person heterogeneity; a high ICC can coexist with clinically important within-person variation. CV is dimensionless and invariant to multiplicative changes of units, but requires a meaningful ratio scale and is unstable when the mean approaches zero; it is generally inappropriate for baseline-subtracted responses that can cross zero. Limits of agreement describe expected differences between repeated measurements but do not prove transportability across contexts. The SD of individual response can be informative in randomized repeated-measures settings, but it is not a direct classifier of individuals and depends on assumptions about variance and measurement error. These quantities should support, not replace, a prespecified model of the response-generating process.

A response phenotype should be re-estimated after major state changes that plausibly alter the response-generating system, such as initiation or discontinuation of glucose-lowering or weight-loss medication, pregnancy, menopause, substantial weight change, acute illness, or major change in physical activity or diet. Importantly, contextual factors counted as part of the phenotype must be specified in advance. Unexplained residual variation should not be relabeled post hoc as “context,” because doing so would make the construct difficult to falsify.

### 4.2. Worked Hypothetical Example: Conditional Postprandial Response

**Figure 1:**
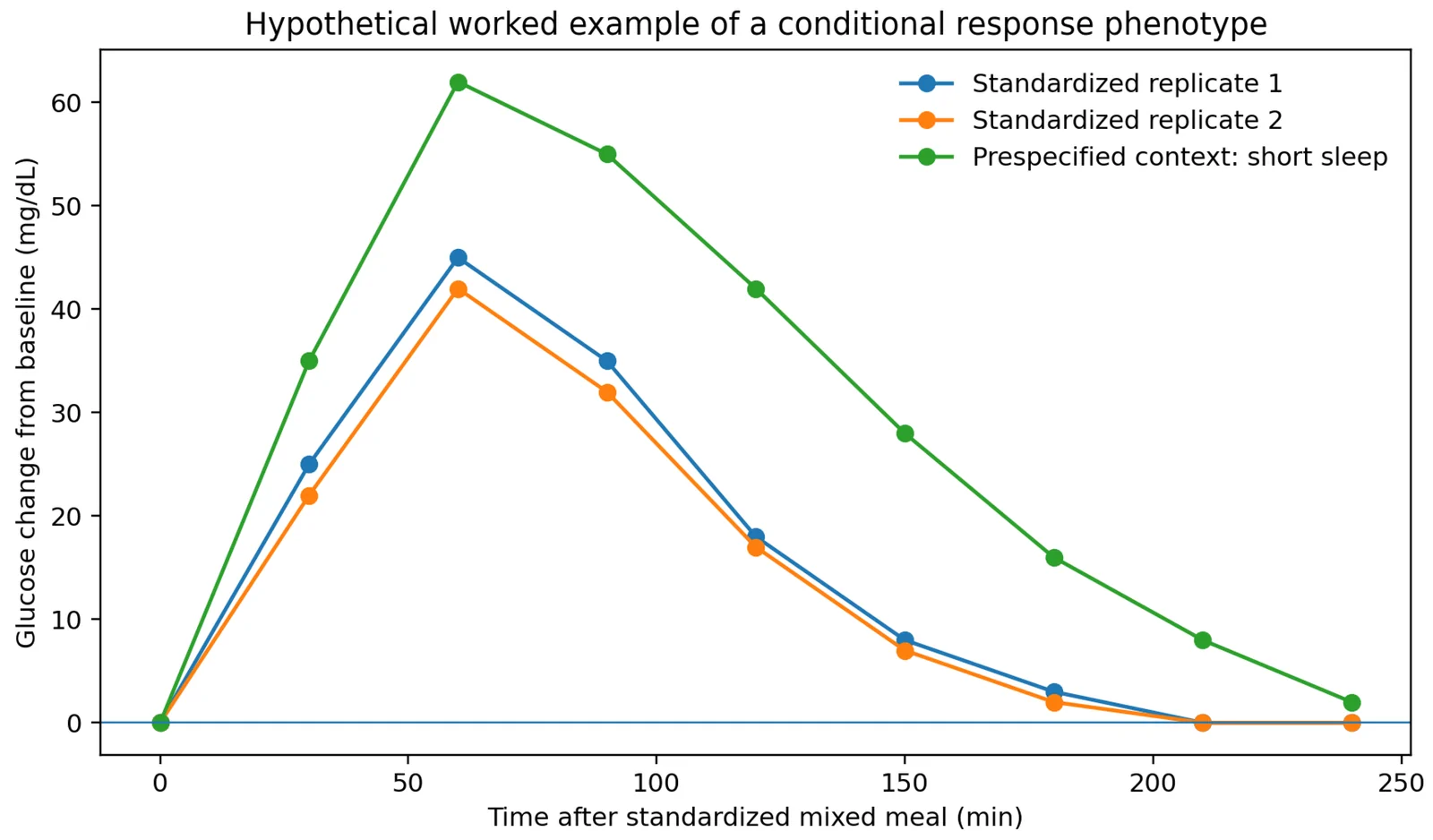
Hypothetical worked example of a conditional response phenotype. The two standardized replicates are similar, whereas a prespecified short-sleep condition produces a larger and slower-resolving glucose excursion. The figure is illustrative and contains no empirical participant data. The curves do not define validated clinical thresholds.

Figure 1 illustrates a hypothetical conditional response phenotype. Consider a prespecified mixed-meal protocol containing a fixed carbohydrate and fat dose, administered twice under standardized conditions and once after a prespecified short-sleep condition. Candidate estimands include glucose incremental area under the curve normalized to carbohydrate dose, peak change from baseline, time to peak, time to return within a prespecified baseline band, triglyceride excursion, and temporal coordination between glucose and insulin. All glucose responses in the illustration are baseline subtracted; because the challenge dose is fixed, additional dose scaling is not required, whereas studies using variable doses would need to prespecify dose normalization. Uncertainty should be represented for each estimand from assay error and repeat variation, and the interpretation is conditional on the challenge definition rather than generalized to all foods or contexts.

In this hypothetical example, two similar replicates suggest possible within-person stability but do not establish reproducibility. Prespecifying sleep as a modifier supports hypothesis testing; a single short-sleep observation cannot establish a sleep effect. To make cross-system discordance explicit, Table 1 assumes that the glucose response worsens after short sleep while the triglyceride trajectory remains within the range of the standardized replicates; this would argue against collapsing the systems into a single adaptive-capacity score. A claim of better or worse adaptive capacity would require a defined external criterion, such as clinically meaningful glycemic exposure, functional performance, symptom burden, or prospective health outcome, together with uncertainty around the component estimands.

Table 2 summarizes selected key studies and the main limits of the inferences relevant to this review.

## 5. Multi-Omics Integration: From Association to Nutritional Decision Utility

Multi-omics is often presented as a direct route to greater precision because genomic, epigenomic, metabolomic, and microbiome layers carry complementary information. The premise is sensible; the integration problem is not simple. Each layer operates on a different temporal scale, has its own error structure, and bears a different relation to causality. Genomic variants are stable but often have modest effects. Epigenetic marks may reflect exposure and regulatory state but depend on tissue and cell composition. Microbiome profiles are compositional and sensitive to geography, medication, and recent diet, whereas metabolomic signals are close to physiology but strongly time dependent [5,6,7,8,31,32,33,34,46]. If these differences are ignored, combining layers can add complexity faster than understanding.

It is helpful to separate descriptive validity, mechanistic plausibility, and decision utility. A biomarker may describe a phenotype reproducibly without being causal. A causal pathway may matter biologically without improving prediction. A predictive feature may improve model performance without identifying an intervention that changes a meaningful outcome. Precision nutrition often moves too quickly across these levels. Current guidance increasingly asks for standardized data generation, replication, external validation, and evidence that a new data layer adds useful information beyond simpler clinical and behavioral variables [5,6,7,8].

Disagreement between layers deserves particular attention. Genetic susceptibility may point in one direction while current metabolomics and clinical function point in another; a microbiome feature may correlate with an exposure but fail to respond to intervention; an epigenetic or miRNA marker may change while functional outcomes remain stable. Such discordance is not automatically a technical failure. It can reflect different temporal scales, compensatory regulation, or the difference between susceptibility and current state. Integration should therefore leave room for disagreement instead of compressing all inputs into a single authoritative score.

Objective characterization of the exposure is as important as characterization of the person. Biomarkers of food intake measured in blood or urine can complement self-report, help assess adherence or misreporting, and support more objective dietary exposure models, although many candidate biomarkers still require validation [47]. Image-assisted dietary assessment, wearable or sensor-assisted recording, and food-metabolome approaches can be combined with conventional dietary methods when the expected reduction in exposure uncertainty justifies the additional burden. These tools should be treated as measurement aids rather than as proof that a particular dietary component caused an observed biological response.

The clinical value of multi-omics should therefore be framed as conditional rather than intrinsically positive or negative. Some multimodal programs have improved a primary outcome and selected secondary outcomes [4], whereas other studies have not shown superiority for their primary endpoint or for the added biological layer [2,3]. Heterogeneity may reflect the information content of the data, but also intervention design, adherence, comparator quality, endpoint selection, assay reliability, and population. The appropriate test is incremental decision value: does the added layer change classification, intervention choice, confidence, safety, or the need for further observation in a way that improves outcomes or reduces burden? If not, simpler clinical, dietary, and behavioral information may be preferable.

## 6. Artificial Intelligence: Prediction, Decision Support, Uncertainty, and Correctability

Artificial intelligence is not a single application class. In precision nutrition, at least five uses should be distinguished: (i) predictive models that estimate an outcome from current features; (ii) temporal models that learn trajectories from repeated measurements; (iii) recommender or decision-support systems that rank or select actions; (iv) digital-twin or mechanistic-hybrid models that simulate alternative states or interventions; and (v) generative AI or large language models (LLMs) that support dietary assessment, communication, summarization, coaching, or interface-level decision support [48,49,50,51,52,53]. These applications have different data requirements, failure modes, and evidentiary standards and should not be treated as interchangeable.

Data complexity alone does not establish that AI is necessary. High-dimensional or multimodal models should be compared with conventional statistical models, parsimonious clinical models, and mechanistic approaches using the same target population, outcome, and evaluation set. Added complexity is justified only if it improves a relevant quantity such as prediction error, calibration, discrimination, decision utility, transportability, or burden. A model that is slightly more accurate but materially less interpretable, more expensive, less transportable, or more difficult to recalibrate may not improve the nutritional decision.

Performance assessment should be matched to the task. For probabilistic prediction, discrimination, calibration-in-the-large, calibration slope, prediction error, and clinically relevant threshold performance may be appropriate. Robustness testing should examine missingness, sensor or assay perturbation, changes in dietary recording, and plausible shifts in input distributions. Transportability requires external evaluation across populations, food environments, ethnic and socioeconomic groups, medication patterns, assay platforms, and devices. Safety assessment should include false reassurance, inappropriate escalation, subgroup performance, and the consequences of acting at proposed thresholds, not only average predictive accuracy [48,49,50,51,52].

Prediction and intervention recommendation are different causal problems. A model can accurately estimate that a person is likely to have a large postprandial response without establishing which alternative intervention would improve that response. Nutritional recommendation requires a defined target population, candidate interventions, a clinically or functionally relevant outcome, an explicit causal question, and evidence about the effects of alternative interventions, ideally from randomized or otherwise causally informative data. Predictive associations may support stratification or monitoring; they should not automatically be interpreted as individualized treatment-effect estimates.

Utility also requires a prespecified action threshold. A well-calibrated model can have little practical value if no threshold changes management, if the recommended action has uncertain benefit, or if the consequences of false-positive and false-negative decisions are poorly defined. Decision-curve approaches or other utility analyses may be useful when thresholds and consequences can be specified, but the more general principle is that model performance should be evaluated against the decision it is intended to change.

Correctability can be operationalized as a sequence rather than a slogan. When observed response diverges materially from a prespecified prediction, the system should verify exposure delivery and measurement quality; assess whether a prespecified context modifier changed; quantify whether the observation lies outside expected uncertainty; reduce confidence when appropriate; test for domain or model drift; recalibrate or revert to a simpler model if performance is no longer supported; and trigger expert review when disagreement affects safety or remains unresolved. A model that cannot revise confidence, document why a recommendation changed, or fall back to a simpler rule is poorly suited to longitudinal individual use.

Generative AI adds a distinct set of risks. LLMs may assist dietary history taking, communication, coaching, summarization, and explanation, but can hallucinate unsupported facts, obscure provenance, express unwarranted confidence, reproduce bias, or generate fluent recommendations that exceed the evidence [53]. Human oversight therefore remains necessary but is not a complete safeguard. Reviewers can themselves be affected by automation bias, overconfidence, alert fatigue, and inappropriate deference to apparently sophisticated outputs. Oversight should consequently include explicit override authority, provenance visibility, escalation rules, and periodic audit of both model and human-review errors.

## 7. Allostasis, Viability, and Adaptive Capacity as Proposed Nutritional Constructs

Several established concepts overlap with the dynamic phenotype described above and should be separated carefully. Metabolic flexibility refers mainly to shifts in substrate use and pathway activity as energetic conditions change. Phenotypic flexibility is an established nutrition-related construct describing coordinated responses across multiple physiological systems to a challenge and has explicitly been framed as adaptive capacity in earlier work [21,23,38]. Resilience emphasizes preservation or recovery of function after perturbation. Allostasis is the established concept of achieving stability through context-sensitive change, whereas allostatic load describes cumulative multisystem burden or the cost of repeated or dysregulated adaptation [54]. These concepts are not introduced by AVP.

In this review, “adaptive capacity” is used more broadly as a candidate latent systems construct rather than as an established clinical endpoint. It is hypothesized to be inferable from repeated, context-specific patterns of response amplitude, timing, cross-system coordination, recovery, and reproducibility. The component features can be measured; their integration into a validated construct of adaptive capacity has not yet been established. Accordingly, the manuscript does not propose a universal adaptive-capacity score or threshold.

**Figure 2:**
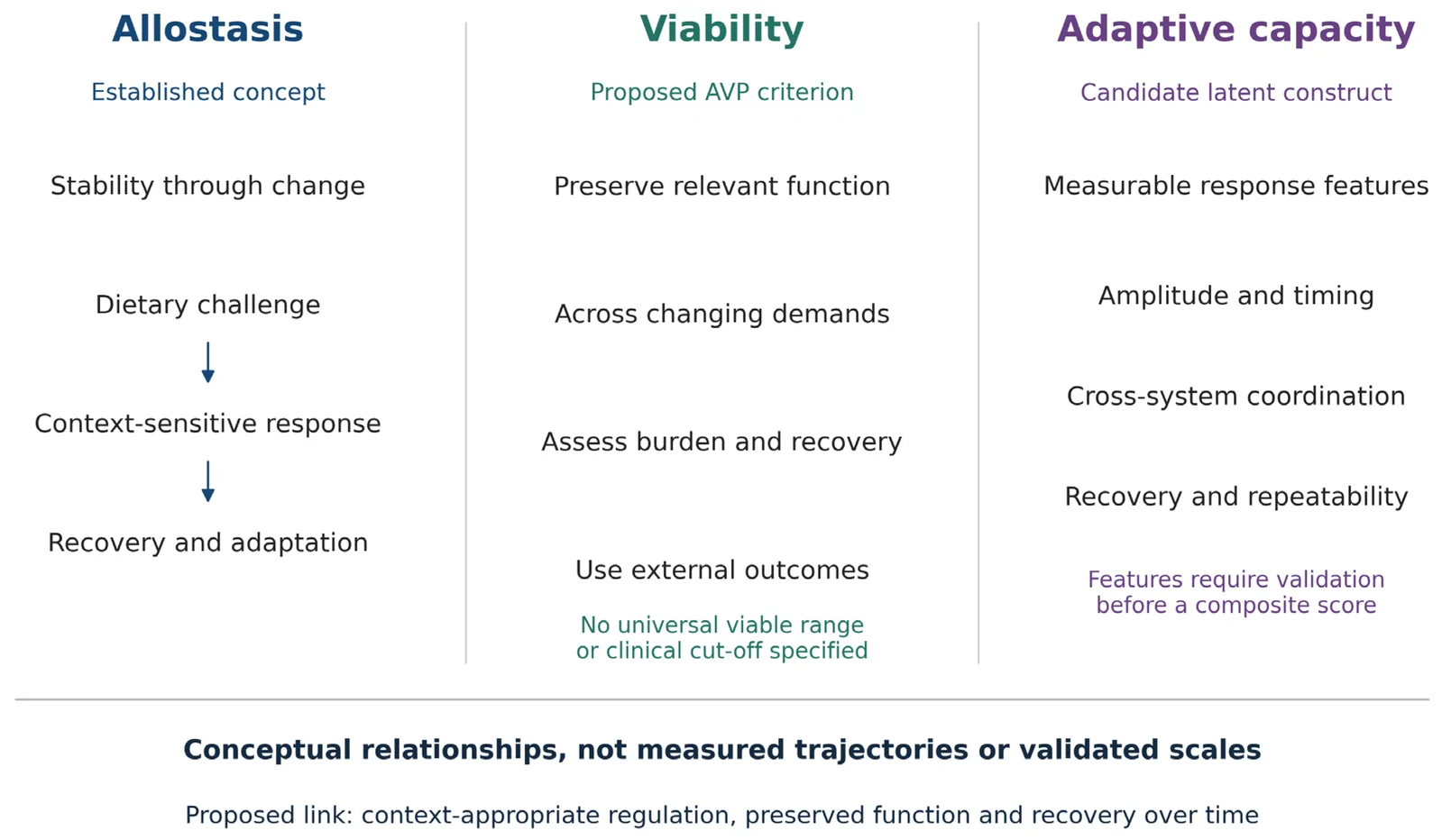
AVP in precision nutrition: allostasis, viability, and adaptive capacity. The conceptual map distinguishes the established concept of allostasis, the proposed AVP criterion of viability, and candidate response features. No empirical trajectories, validated composite score, universal viable range, or clinical threshold are represented. The features require validation against relevant external outcomes before use as a measure of adaptive capacity.

AVP is used here as a conceptual lens linking allostatic adjustment with maintenance of relevant function within a viable range [55]. “Viability” is used in a broad physiological-operational sense rather than as a mathematically formal viability kernel. The proposed interpretation is that an adaptive response should be judged not by minimal deviation from baseline alone, but by whether change is context appropriate, coordinated, compatible with preservation of relevant function, and followed by recovery without disproportionate cost. This interpretation remains hypothesis-generating and requires independent empirical validation (Figure 2).

A substantial excursion from baseline can therefore be adaptive if it is appropriate to the challenge, coordinated with other systems and followed by effective recovery. A small response is not necessarily favorable if it reflects limited reserve, while an excessive or prolonged response can be unfavorable. These statements describe candidate interpretive principles rather than validated individual cut-offs. Their use in precision nutrition requires challenge-specific estimands, repeatability, contextual modeling, and external outcomes that establish whether a response pattern is clinically or functionally meaningful. Table 3 summarizes candidate measurements and evidentiary requirements.

## 8. An AVP-Based Response-and-Revision Model for Adaptive Precision Nutrition

The observations above can be brought together in a decision-oriented model. More molecular data create opportunities for personalization, but they also create more opportunities for error, conflicting signals, and overinterpretation. The starting point should therefore be the decision, not the technology. What information is needed to choose a reasonable intervention and estimate uncertainty? Clinical, dietary, and behavioral information may be enough in many cases. Omics or digital measurements can then be added when there is a plausible expectation that they will change the decision. Complexity becomes an option that is introduced selectively, not a prerequisite for personalization.

Discordant evidence should be handled in the same spirit. Population reference information, an individual’s longitudinal trajectory, molecular measurements, functional outcomes, behavior, and personal goals are different kinds of evidence and should not automatically be collapsed into one score. Agreement across sources can strengthen confidence. Persistent disagreement should do the opposite and may justify further observation, reinterpretation, or expert review. Integration is useful when it clarifies what is known, what remains uncertain, and what could realistically change the next decision.

**Figure 3:**
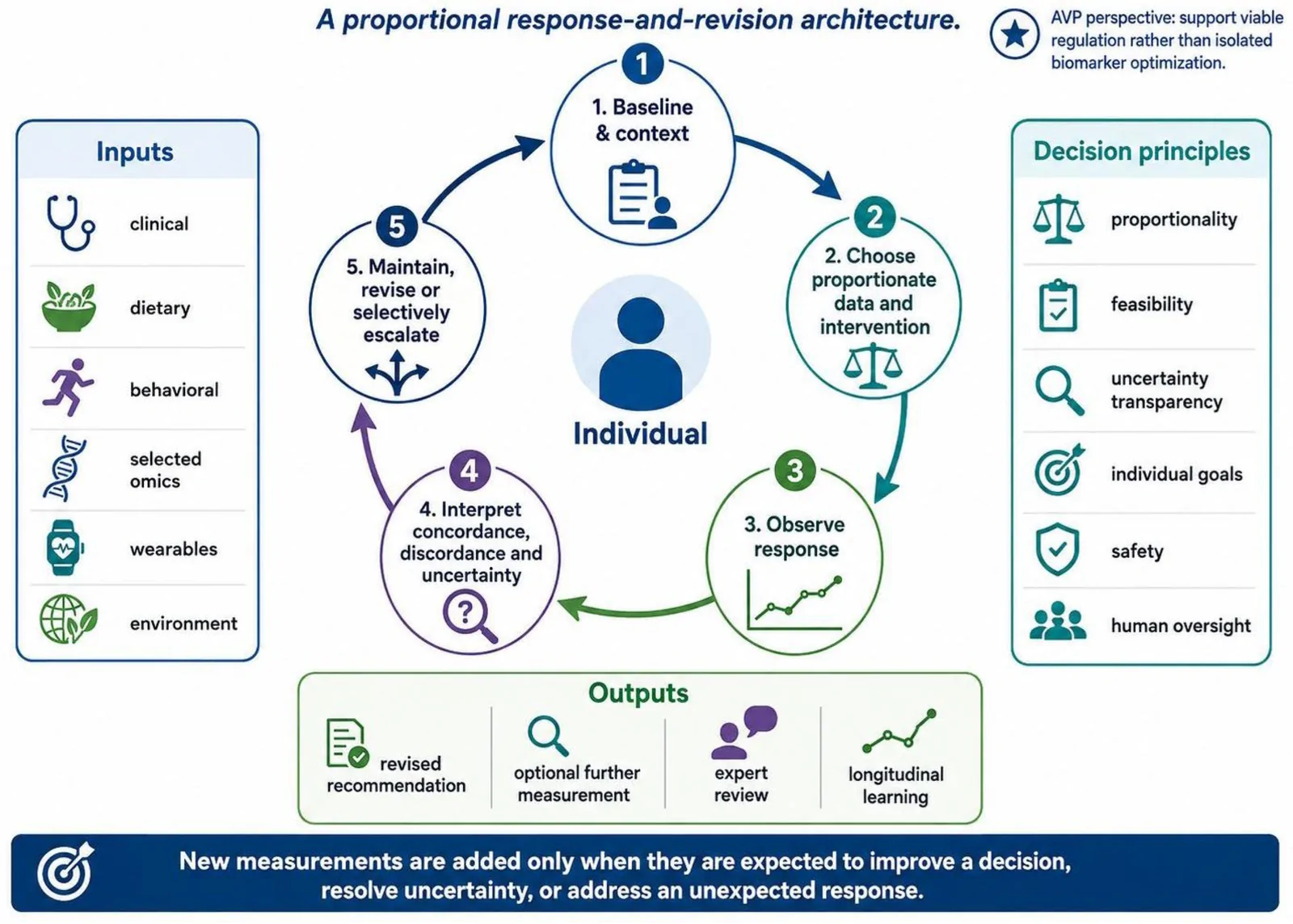
AVP-based adaptive precision nutrition. The diagram illustrates a proposed workflow, not a validated clinical decision algorithm. Arrows indicate intended information flow rather than established causal effects. Proportionality and human oversight guide the operational sequence below.

Adaptive personalized-advice systems already use repeated interaction, behavior, and changing context [13,14]. N-of-1 designs estimate within-person effects; SMARTs develop adaptive treatment sequences; micro-randomized trials estimate proximal effects of time-varying interventions; JITAIs formalize decision points and tailoring variables; phenotypic-flexibility and health-space approaches characterize multidimensional response to challenge; and learning health systems embed feedback from practice into continuing improvement [21,22,23,24,25,26,27,56]. The AVP proposal therefore does not claim novelty for iteration, feedback, repeated measurement, or personalization itself. The proposed additional structure is a physiological criterion of viable regulation plus a proportionality rule that new data should be added only when they can plausibly change a defined decision, reduce relevant uncertainty, or explain an unexpected response. Table 4 compares the approaches, and Figure 3 summarizes the proposed workflow. Box 1 sets out the proposed operational decision sequence.

Box 1 **Proposed operational decision sequence.**

1. Define the target population, nutritional decision, candidate interventions, and clinically or functionally relevant outcome.

2. Establish the minimum clinical, dietary, behavioral, and contextual information needed for a reasonable initial decision.

3. Ask whether causal or trial evidence supports the candidate intervention(s) for the defined target and whether any action threshold is meaningful.

4. Add omics, wearables, biomarkers, or AI only when the added information has a plausible chance of changing intervention choice, safety, confidence, or the need for further observation.

5. Deliver the intervention under sufficiently defined conditions; record prespecified contextual modifiers and exposure/adherence information.

6. Measure the response using prespecified estimands and quantify repeatability, uncertainty, recovery, and concordance with functional or clinical outcomes.

7. Compare expected with observed response. If discordant, verify exposure and measurement, assess prespecified context, reduce confidence where appropriate, and check for model/domain drift.

8. Apply prespecified decision criteria: maintain when the relevant outcome improves beyond expected variation and the intervention remains tolerable; repeat assessment when exposure, context, or measurement uncertainty prevents interpretation; simplify when added data do not alter the decision; recalibrate when verified observations persistently depart from model expectations; selectively add measurements only to answer a defined decision-changing question; and change the intervention or refer when adverse effects, established safety limits, or unresolved clinically important discordance require review.

9. Re-estimate the phenotype after major state changes and document why the next decision differs from the previous one.

These are proposed operational criteria, not validated AVP cut-offs. The relevant outcome, follow-up interval, acceptable uncertainty, and safety thresholds must be specified for the target population and intervention; a change in an exploratory molecular marker alone does not establish benefit. Table 5 distinguishes supporting observations from the inferences they permit.

**Table 5:** Key supporting observations and the limited inference they support.

Observation from Literature	Supported Inference	Not Established by That Observation Alone	Framework Implication
Personalized advice can improve behavior or selected outcomes	Personalization can influence behavior/outcomes	Added omics caused the benefit	Test incremental value; proportionality
Inter-individual postprandial heterogeneity	People differ in measured responses	Stable individual responder phenotype	Require replication and person-by-food evidence
Repeated responses vary with sleep, activity, order or context	Context can systematically alter response	All unexplained variation is meaningful context	Prespecify modifiers; model conditional phenotype
miRNA, metabolite, or microbiome signals change	Biological activity or association	Clinical or functional benefit	Outcome hierarchy
Prediction accuracy varies across cohorts/platforms	Transportability is limited	AI is necessarily superior or inferior	External validation, recalibration, fallback
Prediction and observed response disagree	Model/exposure/context uncertainty exists	Which intervention will causally improve the outcome	Correctability and causal evidence before recommendation

The outcome hierarchy is essential. Molecular changes may be early and mechanistically informative, but they should not automatically outrank established clinical or functional endpoints. In the combined probiotic-octacosanol pilot intervention discussed above, selected miRNA and inflammatory transcripts changed while anthropometric and lipid variables did not [35]. This suggests biological activity of the combined intervention, not by itself a health benefit. The reverse is also possible: a clinically meaningful benefit need not be accompanied by a striking change in a favored molecular marker. Interpretation should rest on relevance, concordance, temporal relation, and the evidentiary level of the outcome rather than on assay sophistication.

Feasibility and individual goals belong in the same assessment. A biologically plausible strategy that is chronically incompatible with a person’s food environment, preferences, resources, or schedule has limited practical validity. Proportionality follows from the same logic. Repeated phenotyping and multi-omics can be expensive and burdensome, and more monitoring can reduce adherence. A practical system should begin with the least complex information sufficient for a meaningful decision and add measurements only when their expected decision value justifies cost, burden, and inequity risk [5,13,14,52,57,58].

The component that genuinely depends on the AVP proposal is therefore narrow: when several legitimate evidence sources and adaptive methods are available, AVP proposes that the decision target be framed as preservation of viable, context-appropriate regulation rather than optimization of isolated biomarkers, and that measurement escalation be proportional to decision value. A standard iterative-care model could make many of the same practical decisions. The AVP hypothesis becomes scientifically meaningful only if those explicit criteria lead to different, reproducible, and ultimately better decisions in comparative studies.

## 9. Research Priorities, Limitations, and Translational Implications

The AVP-based formulation is an open, hypothesis-generating framework rather than a validated clinical system. Its value depends on whether dynamic response information, selected molecular layers, or AI-supported integration add useful information beyond high-quality clinical, dietary, and behavioral assessment. The relevant validation question is not merely whether response phenotypes can be detected or predicted, but whether the response-and-revision architecture improves a prespecified clinical or functional outcome, decision quality, burden, adherence, safety, or cost compared with a strong simpler comparator.

Prospective evaluation should therefore define the target population, decision, intervention options, outcome hierarchy, and action thresholds before model development or deployment. A practical study could randomize participants to high-quality personalized nutrition based on conventional clinical/dietary information versus the same care plus AVP-organized dynamic response and selective measurement. Primary outcomes should be clinical or functional where feasible; secondary outcomes could include calibration, rate of recommendation change, number and cost of additional tests, adherence, patient burden, adverse events, uncertainty resolution, and frequency of appropriate simplification or referral. To distinguish the value of the AVP organizing criteria from the effects of additional measurements or contact, a matched-information adaptive comparator or factorial design would also be needed. Improved prediction alone would not establish clinical utility.

Dynamic response features themselves require validation. Candidate features such as amplitude, time to peak, recovery, coordination, and context sensitivity should first demonstrate adequate measurement reliability and within-person stability for the intended use. External validity then requires testing whether the same feature retains meaning across free-living conditions, populations, food environments, and measurement platforms. The response phenotype may need recalibration after medication initiation, use of GLP-1 receptor agonists, or other agents that alter appetite and gastric or metabolic responses, pregnancy, menopause, major weight change, acute illness, or material changes in sleep, activity, or environmental conditions.

Model drift and domain shift are especially relevant for AI-supported systems. Food availability, culture, assay platforms, wearable firmware, clinical practice, and population case-mix can change over time. An operational system should monitor input distribution and calibration, define triggers for recalibration, document version changes, and permit reversion to a simpler validated rule when evidence for the complex model is insufficient. Human override should be explicit and auditable: the reviewer should be able to reject a model recommendation, state the reason, and trigger further observation, but oversight processes should also be monitored for automation bias, overconfidence, alert fatigue, and inconsistent overrides.

NPH and PREDICT-like infrastructures provide relevant test environments because they combine controlled or standardized nutritional exposures with contextual and multi-omic data and predictive modeling [7,11,12]. AVP would add a testable decision layer rather than another measurement layer: Does the added information cross a prespecified threshold for changing the next intervention, confidence, or need for further measurement? Does the response then support or weaken the prior interpretation? And does using this loop improve outcomes compared with a simpler model? These questions make the proposal falsifiable.

Several limitations remain. A narrative synthesis is vulnerable to selection and interpretation bias and does not provide formal pooled estimates. The proposed construct of adaptive capacity is not independently validated, and the primary AVP reference is the author’s own recent conceptual work [55]. The present manuscript therefore cannot establish that viable regulation is a superior physiological target or that an AVP-organized system improves care. It can only define the proposal, distinguish it from adjacent frameworks, identify measurable components, and specify comparisons that would support or falsify its utility.

## 10. Conclusions

Precision nutrition has reached a stage at which greater measurement capacity does not automatically imply better decisions. The literature supports closer attention to reproducible, context-dependent responses to nutritional challenges and interventions, while also showing that molecular activity, prediction accuracy, and clinical utility are distinct evidentiary levels. The proposed response-and-revision architecture treats an initial recommendation as a provisional hypothesis: baseline information guides an intervention; observed response, recovery, feasibility, repeatability, uncertainty, and outcome concordance are assessed; and the next decision may be maintained, simplified, recalibrated, changed, selectively escalated, or referred.

AVP is proposed here as a hypothesis-generating conceptual framework for organizing this process around viable, context-appropriate regulation, and proportional use of additional measurements. It does not replace phenotypic flexibility, N-of-1 methods, adaptive personalized-advice systems, causal intervention evidence, or clinical endpoints, and it is not independently validated. The strongest conclusion currently supported is therefore organizational rather than clinical: the framework provides a testable structure for asking when additional individual-level information is reproducible, relevant, and sufficiently decision changing to justify its use. Prospective comparison with high-quality simpler approaches is required before any claim of superior nutritional or clinical outcomes is warranted.

## Figures and Tables

**Table 1:** Hypothetical values accompanying Figure 1. Values are illustrative and are not validated measurements or clinical cut-offs.

Condition	Peak Change	Recovery	Repeatability/Uncertainty /Cross-System Context	Decision Implication
Standardized replicate 1	+45 mg/dL at 60 min	~210 min	Reference replicate; assay error and repeat variation still need quantification	No responder label from one exposure
Standardized replicate 2	+42 mg/dL at 60 min	~210 min	Peak differs by 3 mg/dL; close replication; triglyceride trajectory also assumed similar	Suggests possible within-person stability
Short sleep (prespecified)	+62 mg/dL at 60 min	>240 min	Glucose change exceeds replicate spread; triglyceride remains within replicate range (illustrative discordance)	Model sleep as modifier; do not call it random noise

**Table 2:** Selected key studies and their limitations. The selection illustrates distinct evidentiary questions and is not an exhaustive evidence grading.

Study	Design and Scope	Main Finding	Main Limitation
Food4Me [2]	Randomized advice; 6 months	Dietary behavior improved; added phenotype/genotype did not show further benefit.	Tested advice and data layers; not all possible omics strategies.
Popp et al. [3]	Randomized trial; 6 months	PPGR-targeted diet did not outperform low-fat diet for weight loss.	Endpoint-specific null result; adherence and exposure matter.
ZOE METHOD [4]	Randomized program; 18 weeks	Primary triglyceride outcome improved; primary LDL-C outcome did not.	Bundled intervention cannot isolate each personalization layer.
PREDICT 1 [11]	Challenge/prediction study; external cohort	Large variability; prediction performance differed by outcome.	Prediction does not establish benefit from using the model.
Zhang et al. [22]	Repeated n-of-1 trials; independent study	Individual glycemic sensitivity showed temporal consistency.	Sensitivity is not equivalent to food-specific interaction or clinical utility.
Della Corte et al. [43]	Secondary analysis; 382 healthy adults	GI scaling explained responses within test-retest variability.	Standardized testing of nine foods; not all populations or contexts.
Wu et al. [44]	Replicated challenges; 55 participants	Food-response patterns associated with metabolic physiology.	Small mechanistic study; no test of clinical decision utility.

**Table 3:** Candidate dynamic response features and minimum evidentiary requirements for proposed operationalization of adaptive capacity.

Challenge/ Context	Candidate Measurements	Dynamic Feature	Minimum Repeatability/Stability	Key Context/ Confounders	Decision-Oriented Interpretation
Standardized mixed meal	Glucose, insulin, triglycerides, metabolomics, CGM	Amplitude, time to peak, coordination, recovery	At least repeated standardized exposure; quantify within-person variation and measurement error	Meal matrix/dose, fasting, prior meal, sleep, activity, medication, sensor/assay	Is the response stable enough under defined conditions to inform a decision?
Repeated food exposure	Postprandial glucose/lipids, symptoms, exposure markers	Person-by-food interaction, day-to-day variance	Replicate exposures to both test and comparator conditions where feasible	Order, co-ingestion, recent diet, adherence, acute illness	Does reproducible person-by-food sensitivity exceed contextual and stochastic variation?
Functional-food intervention	Exposure metabolites, microbial metabolites, regulatory RNAs, functional outcomes	Concordance across exposure, molecular response, function, recovery	Replicate exposure or longitudinal confirmation when individual classification is intended	Product composition, batch, dose, microbiome, medication, adherence	Does biological activity translate into functional or clinical benefit?
Fasting/meal timing/substrate transition	Glucose, ketones, oxidation, flux estimates	Switching rate, recruitment, recovery after refeeding	Repeat challenge or stable longitudinal pattern	Sleep/circadian timing, exercise, recent diet, medications	Is substrate switching reproducible and relevant to a defined outcome?
Longitudinal dietary intervention	Clinical measures, wearables, adherence, selected omics, patient goals	Trajectory, adaptation, persistence, discordance	Repeated outcome assessment with prespecified recalibration rules	Weight change, medication, life stage, illness, season/environment	Maintain, simplify, recalibrate, escalate or refer?

**Table 4:** Comparison of the proposed AVP framework with related established approaches, including allostasis/allostatic load, multi-omics precision nutrition, and AI-supported prediction or decision support. The table is descriptive and does not rank the approaches.

Approach	Primary Unit/Input	Time & Repeated Response	Uncertainty/ Statistical Testing	Adaptation Mechanism	Typical Outcome Emphasis	Relation to Proposed AVP
Conventional personalized nutrition	Baseline individual characteristics	Usually limited longitudinal updating	Varies; often population evidence plus tailoring	Advice adapted to baseline traits/preferences	Behavioral and clinical outcomes	AVP adds no new baseline personalization; proposes explicit response/revision and proportional data use
PhenFlex/challenge testing	Multisystem response to standardized perturbation	Central; response and recovery trajectories	Challenge-specific reproducibility and multivariate response	Interpretation of dynamic physiological flexibility	Dynamic biomarkers/health-related response	AVP adopts dynamic response logic but adds decision proportionality and explicit outcome hierarchy
Health-space models	Multidimensional biological processes	Can represent treatment-response movement	Multivariate modeling/process scores	Visualization/interpretation of movement in biological space	Multidimensional molecular/physiological response	AVP’s viable zone is conceptually adjacent; AVP proposes a decision rule rather than a new visualization method
Repeated N-of-1	Individual under repeated intervention/control periods	Central	Within-person effect estimation, period/carryover considerations	Select intervention based on individual comparative effect	Individual treatment effect for defined outcome	AVP can use N-of-1 evidence but adds cross-layer hierarchy, proportional measurement and broader correctability
SMART	Sequential treatment strategy	Repeated decision stages	Randomized estimation of adaptive treatment sequences	Continue/augment/switch based on intermediate response	Optimized adaptive treatment strategy	AVP does not replace SMART; it proposes physiological and measurement criteria that could inform decision rules
MRT/JITAI	Momentary decision points and tailoring variables	High-frequency repeated context	Randomized proximal effects/time-varying moderators	Deliver or withhold intervention at opportune times	Proximal behavioral or health response	AVP is less about delivery timing and more about interpretation of physiological response and data proportionality
Adaptive personalized-advice systems	Repeated behavior/context feedback	Central	Depends on implementation	Advice updated with behavior/context	Behavior, adherence, dietary goals	Closest comparator; AVP proposes viable regulation and explicit outcome hierarchy as additional organizing criteria
Learning health systems	Patient/practice data across system	Continuous learning at system level	Real-world evaluation/implementation methods	Feedback from care to knowledge to care	Quality, safety, value, outcomes	AVP is individual/physiology focused rather than organizational; shares feedback/correctability logic
Allostasis/allostatic load	Multisystem regulatory adjustment and accumulated physiological burden	Allostasis is dynamic; allostatic load reflects cumulative burden over time	Indices and longitudinal models vary; no single universal measure	Stability through change; dysregulated or repeated adaptation may accumulate cost	Context-sensitive regulation/multisystem burden	AVP uses allostasis as established physiological background but adds the proposed viability criterion and proportional decision rule; it does not replace allostatic-load concepts
Multi-omics precision nutrition	Integrated genomic, epigenomic, metabolomic, microbiome and related molecular layers	Often baseline-focused, but can be longitudinal	Layer-specific error, integration uncertainty, external validation, and incremental-value testing	Usually prediction, stratification, or mechanistic interpretation rather than an intrinsic feedback rule	Molecular profiles, prediction and mechanistic hypotheses	AVP treats omics as optional data layers that must justify incremental decision value rather than as a prerequisite for personalization
AI-supported prediction/decision support	Clinical, dietary, digital, and/or molecular data processed by statistical or machine-learning models	May be static or explicitly temporal depending on the application	Discrimination, calibration, robustness, subgroup performance, transportability, and model/domain drift	Prediction, recommendation, simulation or updating depending on the model and causal evidence	Prediction or decision support; utility depends on the action threshold and intervention evidence	AVP adds no new algorithm; it requires that AI-supported recommendations expose uncertainty, meet causal/action-threshold requirements, and remain correctable or revertible
Proposed AVP-based framework	Defined nutritional decision plus minimum necessary individual data	Repeated response/recovery only when decision-relevant	Visible uncertainty, reproducibility, concordance, recalibration	Maintain, simplify, recalibrate, selectively escalate, or refer	Clinical/functional outcomes prioritized over isolated molecular change	Hypothesis-generating synthesis to be tested against simpler and established adaptive approaches

## Data Availability

No new data were created or analyzed in this study. Data sharing is not applicable to this article. Search queries, retrieved identifiers, and bibliographic records for the targeted supplementary search were retained by the author.

## Abbreviations

AI, artificial intelligence; AVP, Allostatic Viability Principle; CGM, continuous glucose monitoring; CV, coefficient of variation; GI, glycemic index; ICC, intraclass correlation coefficient; LDL-C, low-density lipoprotein cholesterol; JITAI, just-in-time adaptive intervention; LLM, large language model; MRT, micro-randomized trial; NPH, Nutrition for Precision Health; PN, precision/personalized nutrition; PPGR, postprandial glycemic response; SD, standard deviation; SMART, sequential multiple assignment randomized trial.

## Appendix A. Supplementary Search Strategy

PubMed searches executed on 3 October 2026. All six queries used the publication-date restriction shown below. Counts are retrieved records before cross-query deduplication; 804 occurrences corresponded to 745 unique PMIDs. Additional named-study and DOI retrieval was recorded separately. This was a targeted narrative update, not exhaustive eligibility screening.

S1 (356 records): ((“personalized nutrition”[Title/Abstract] OR “personalised nutrition”[Title/Abstract] OR “precision nutrition”[Title/Abstract] OR “personalized diet”[Title/Abstract]) AND (randomized controlled trial[Publication Type] OR randomised[Title/Abstract] OR randomized[Title/Abstract])) AND (“2021/01/01”[Date—Publication]: “2026/10/03”[Date—Publication])

S2 (33 records): ((“postprandial”[Title/Abstract] OR “glycemic response”[Title/Abstract]) AND (repeatability[Title/Abstract] OR reproducibility[Title/Abstract] OR “person-specific”[Title/Abstract] OR “n-of-1”[Title/Abstract]) AND (nutrition[Title/Abstract] OR food[Title/Abstract] OR meal[Title/Abstract] OR dietary[Title/Abstract])) AND (“2021/01/01”[Date—Publication]: “2026/10/03”[Date—Publication])

S3 (102 records): ((“phenotypic flexibility”[Title/Abstract] OR “adaptive personalized nutrition”[Title/Abstract] OR “allostatic viability”[Title/Abstract])) AND (“2021/01/01”[Date—Publication]: “2026/10/03”[Date—Publication])

S4 (113 records): ((“precision nutrition”[Title/Abstract] OR “personalized nutrition”[Title/Abstract]) AND (“external validation”[Title/Abstract] OR “clinical utility”[Title/Abstract] OR “incremental”[Title/Abstract] OR “decision”[Title/Abstract])) AND (“2021/01/01”[Date—Publication]: “2026/10/03”[Date—Publication])

S5 (119 records): ((“functional food”[Title/Abstract] OR polyphenol[Title/Abstract] OR probiotic[Title/Abstract]) AND (“personalized nutrition”[Title/Abstract] OR metabotype[Title/Abstract] OR “individual response”[Title/Abstract])) AND (“2021/01/01”[Date—Publication]: “2026/10/03”[Date—Publication])

S6 (81 records): ((“precision nutrition”[Title/Abstract] OR “personalized nutrition”[Title/Abstract]) AND (“systematic review”[Publication Type] OR “meta-analysis”[Publication Type])) AND (“2021/01/01”[Date—Publication]: “2026/10/03”[Date—Publication])

Supplementary retrieval used the DOI queries 10.1093/ije/dyw186[doi], 10.1038/s41591-020-0934-0[doi], and 10.1038/s41467-025-66498-x[doi], and direct retrieval of PMIDs 34301736 and 40467897 after named-study identification. No date restriction was applied to these supplementary retrievals.
